# Sarcoptic mange in the Scandinavian wolf *Canis lupus* population

**DOI:** 10.1186/s12917-016-0780-y

**Published:** 2016-07-27

**Authors:** Boris Fuchs, Barbara Zimmermann, Petter Wabakken, Set Bornstein, Johan Månsson, Alina L. Evans, Olof Liberg, Håkan Sand, Jonas Kindberg, Erik O. Ågren, Jon M. Arnemo

**Affiliations:** 1 Faculty of Applied Ecology and Agricultural Sciences, Hedmark University College, Campus Evenstad, N-2480 Koppang, Norway; 2 Department of Virology, Immunobiology and Parasitology, National Veterinary Institute, SE-75189 Uppsala, Sweden; 3 Department of Ecology, Grimsö Wildlife Research Station, Swedish University of Agricultural Sciences, SE-73091 Riddarhyttan, Sweden; 4 Department of Wildlife, Fish and Environmental Studies, Swedish University of Agricultural Sciences, SE-90183 Umeå, Sweden; 5 Department of Pathology and Wildlife Disease, National Veterinary Institute, SE-75189 Uppsala, Sweden

**Keywords:** *Canis lupus*, Grey wolf, *Sarcoptes scabiei*, Sarcoptic mange, Ectoparasites, ELISA, Red fox, *Vulpes vulpes*, Wildlife disease

## Abstract

**Background:**

Sarcoptic mange, a parasitic disease caused by the mite *Sarcoptes scabiei,* is regularly reported on wolves *Canis lupus* in Scandinavia. We describe the distribution and transmission of this parasite within the small but recovering wolf population by analysing 269 necropsy reports and performing a serological survey on 198 serum samples collected from free-ranging wolves between 1998 and 2013.

**Results:**

The serological survey among 145 individual captured Scandinavian wolves (53 recaptures) shows a consistent presence of antibodies against sarcoptic mange. Seropositivity among all captured wolves was 10.1 % (*CI.* 6.4 %–15.1 %). Sarcoptic mange-related mortality reported at necropsy was 5.6 % and due to secondary causes, predominantly starvation. In the southern range of the population, seroprevalence was higher, consistent with higher red fox densities. Female wolves had a lower probability of being seropositive than males, but for both sexes the probability increased with pack size. Recaptured individuals changing from seropositive to seronegative suggest recovery from sarcoptic mange. The lack of seropositive pups (8–10 months, *N* = 56) and the occurrence of seropositive and seronegative individuals in the same pack indicates interspecific transmission of *S. scabiei* into this wolf population.

**Conclusions:**

We consider sarcoptic mange to have little effect on the recovery of the Scandinavian wolf population. Heterogenic infection patterns on the pack level in combination with the importance of individual-based factors (sex, pack size) and the north–south gradient for seroprevalence suggests low probability of wolf-to-wolf transmission of *S. scabiei* in Scandinavia.

## Background

Sarcoptic mange is an epizootic skin disease caused by the mite *Sarcoptes scabiei* worldwide infesting over 100 mammalian hosts including wild and domestic canids [1, 2]. The mite, burrowing through the stratum corneum, causes the host to mount a humoral immunological response [3–5]. Wolves *Canis lupus* infested by *S. scabiei*, develop alopecia due to intense scratching and biting triggered by a hypersensitive response and may become debilitated and emaciated due to secondary bacterial infections and difficulties in catching the natural prey [1, 2, 6]. *S. scabiei* infections can reduce pack size, annual pack growth rate and cause additive mortality [7, 8]. However, recovery from even severe sarcoptic mange on wolves is reported from northern Spain and Yellowstone National Park [7, 9].

*S. scabiei* actively seek olfactory and thermal stimuli and are able to survive, in suitable environments, for up to 19 days off the host. All life stages remain infective for at least one-half to two-thirds of their survival time [10]. Transmission normally occurs through close contact between hosts and is assumed to be host-density dependent [1] but also fomites in the host environment can be a source of transmission [10]. In Yellowstone National Park, the spatio-temporal patterns of *S. scabiei* infestation on wolves are related to distance to the next infested pack, indicating wolf-to-wolf transmission [7]. In Scandinavia interspecific transmission of *S. scabiei* var. *vulpes* from red fox *Vulpes vulpes* is the most likely origin for mange in wolves, domestic dog *Canis lupus familiaris,* arctic fox *Alopex lagopus,* lynx *Lynx lynx* and domestic cat *Felis catus* [11, 12]. In northern Spain, wolves are infested with *S. scabiei* originated from both red foxes and ungulates, emphasising the prey-to-predator transmission [13].

The wolf was regarded as functionally extinct in Scandinavia during the late 1960’s. In 1983, two immigrant wolves from the Finnish-Russian wolf population reproduced for the first time and became the founders of the present Scandinavian wolf population, [14–16]. During the following 30 years, the population increased from less than 10 individuals to an estimated size of approximately 400 wolves [17]. By 2013, only five Finnish-Russian founders had genetically contributed to this population, and severe inbreeding depression has been confirmed [15]. Although mortality of Scandinavian wolves is mainly human-caused [18], sarcoptic mange may be an important cause of natural mortality [11]. Sarcoptic mange arrived in Scandinavia in the mid-1970’s with devastating effects on the red fox population, [19, 20]. A previous study focusing on immunoglobulin E (IgE) levels found 14/57 Scandinavian wolves seropositive for sarcoptic mange [21]. Effects of sarcoptic mange on the demography of the Scandinavian wolf population remain unclear but have the potential to influence this small, inbred population. Here we analyse and present an overview of the distribution of sarcoptic mange in the Scandinavian wolf population.

Between 1998 and 2013 a total of 198 serum samples from live wolves were collected and analysed by two different enzyme-linked immunosorbent assays (ELISA) and complemented by Western Blot. In addition, we evaluated necropsy reports of 269 dead wolves collected in Sweden between 2003 and 2013.

The aim of our study is to describe the occurrence of sarcoptic mange in the Scandinavian wolf population and to identify demographic and environmental factors that relate to the probability of finding seropositive samples. Based on the literature and personal observations we expected sarcoptic mange to be a minor threat to the Scandinavian wolf population. We predicted the probability of sarcoptic mange occurrence to depend on population level factors including red fox and wolf territory density rather than on individual-based factors such as age and sex of wolves.

## Results

### Seropositivity on captured wolves

**Fig. 1:**
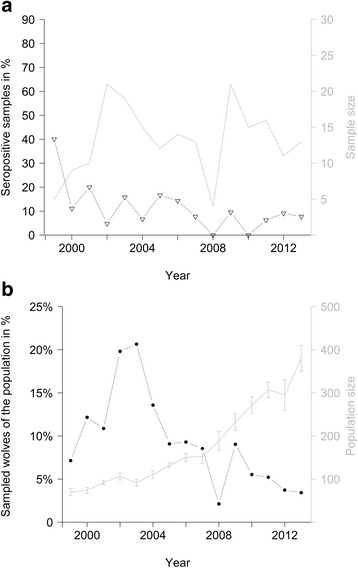
The proportion of seropositive samples (**a**) and the sample size as the percentage of the Scandinavian wolf population (**b**). Triangles (**a**) show the proportion of seropositive samples to the sampled number of wolves each year (grey line). Solid dots (**b**) show the percentage of sampled wolves. The grey line (**b**) represents the mean number of individual wolves reported in Scandinavia with error bars representing minimum and maximum estimations [17]

In total 178 of the 198 samples (89.9 %) (Tables 1 and 2) were tested seronegative and 20 samples (10.1 %, Wilson 95 % *CI* 6.4 %–15.1 %) were tested seropositive. Mean annual proportion of seropositive samples was 11.3 % (SE 2.5 %), ranging from 40.0 % in 1999 (*N* = 5) to zero in 2008 (*N* = 7) and 2010 (*N* = 15) (Fig. 1a). The annual proportion of the wolf population that was sampled decreased during the study period (χ_1,13_ = 18.82; *p* < 0.01) (Fig. 1b). The annual sample size did not allow for further temporal analysis.

**Table 1:** Demographic distribution of the serum samples and observed lesions indicating sarcoptic mange among the captured individuals

		Total number of serum samples	Seropositive serum samples
Pups	Pups total	56	0
	Females	28	0
	Males	28	0
	Single/Dispersing	0	0
	Pair	0	0
	Pack	56	0
	Unclear pack structure	0	0
	Alopecia reported	0	0
Adults	Adults total	142	20
	Females	66	6
	Males	76	14
	Single/Dispersing	11	1
	Pair	54	6
	Pack	71	13
	Unclear pack structure	6	1
	Alopecia reported	9	7

Pups are < 1 year old, adults > 1 year old. Single/Dispersing wolves are outside the parental territory and have no territory established yet. Pairs are a male and a female in an established territory. Packs are one or two reproducing wolves with their < 2 year old offspring. Alopecia reported on the capture form

**Table 2:** Results from ELISA and Western Blot analysis

ID Wolf	Sampling date	Sex	Age class	OD MSA – 1	OD Crude	Result ELISAs	Western Blots
9802	15.12.1998	Male	Adult	0.71	0.83	++	++
9803	15.12.1998	Female	Adult	0.73	0.91	++	+
9808	27.01.2000	Male	Adult	0.11	0.32	−+	--
0001	28.01.2000	Male	Adult	1.54	2.18	++	++
0002	28.01.2000	Female	Adult	0.11	0.09	--	-
0003	28.01.2000	Female	Juvenile	0.05	0.07	--	-
0104	10.02.2001	Female	Adult	1.14	1.20	++	++
0105	10.02.2001	Male	Adult	1.66	1.96	++	++
0105	05.02.2003	Male	Adult	0.76	0.73	++	+
0109	12.02.2001	Male	Adult	0.63	0.11	+−	-
0109	08.12.2001	Male	Adult	0.34	0.13	--	-
0106	12.02.2001	Female	Juvenile	0.11	0.14	--	-
0111	21.12.2001	Female	Adult	0.20	0.30	-?	+
0203	13.01.2002	Female	Adult	0.17	0.24	-?	-
0208	26.01.2002	Male	Adult	0.41	0.15	?-	-
0208	06.03.2003	Male	Adult	1.32	1.30	++	+
0215	01.02.2002	Male	Adult	0.20	0.25	-?	-
0214	05.03.2003	Male	Adult	0.70	0.92	++	+
0214	21.01.2004	Male	Adult	0.41	0.65	?+	+
0307	06.03.2003	Female	Adult	0.15	0.23	-?	-
0009	23.01.2004	Male	Adult	0.36	0.13	?-	-
0504	14.03.2005	Male	Adult	0.40	0.42	?+	+
0506	16.03.2005	Male	Adult	0.56	0.19	+−	?+
0506	09.03.2007	Male	Adult	0.43	0.07	?-	-
0507	16.03.2005	Female	Juvenile	0.26	0.15	--	-
0507	08.02.2008	Female	Adult	0.65	0.13	+−	-
0601	27.01.2006	Female	Adult	0.44	0.37	?+	-
0606	01.02.2006	Male	Adult	0.22	0.36	−+	+
0606	08.03.2007	Male	Adult	0.12	0.20	--	
0611	13.02.2006	Male	Adult	0.22	0.25	-?	+
0611	11.03.2007	Male	Adult	0.43	0.21	??	-
0704	07.03.2007	Female	Adult	0.67	0.70	++	+
0913	30.01.2009	Male	Juvenile	0.10	0.35	−+	-
0916	11.02.2009	Male	Adult	1.50	0.40	++	+
0918	12.02.2009	Male	Adult	1.01	0.80	++	+
0918	11.02.2010	Male	Adult	0.23	0.19	--	
1004	10.02.2010	Female	Juvenile	0.18	0.22	-?	-
1114	21.03.2011	Female	Adult	0.73	0.39	++	+
1114	11.12.2011	Female	Adult	0.98	0.37	++	-
1114	06.02.2012	Female	Adult	0.81	0.35	++	?-
1114	14.03.2013	Female	Adult	1.19	0.37	++	+
1202	16.12.2011	Male	Adult	1.54	0.98	++	+

OD MSA-1: Relative optical densities using the MSA-1 antigen in the ELISA, cut off are 0.35 (doubtful) and 0.499 (seropositive) respectively. OD Crude: Relative optical densities using the crude antigen in the ELISA, cut off 0.2 (doubtful) and 0.299 (seropositive). Result ELISAs are corresponding to the OD values and decide between: + (positive), − (negative) and ? (doubtful). Shown are all samples with either positive or doubtful results from one of the ELISA and five random selected with negative results

Among the 38 recaptured individuals, eight were tested seropositive at least once. Six were seropositive at first capture and four seronegative at recapture 1 year later. Two individuals were seropositive both at first capture and at recapture 2 years later (Table 2).

In 61 of 95 territory sampling events, multiple wolves were captured within the same territory. In 13 of these 61 sampling events, at least one individual was seropositive and there were always also seronegative wolves in the same sampling event. In two family groups, both the adult male and female tested seropositive but their captured pups (two each) were seronegative at time of capture. In eight territories, seropositive individuals were recaptured and six tested seronegative at recapture. In one territory, both the adult male and female were captured in two consecutive years, the male tested seropositive twice and the female seronegative twice.

### Sarcoptic mange from wolves at necropsy

The average annual prevalence of sarcoptic mange among dead wolves collected in Sweden between 2003 and 2013 was 4.5 % (SE = 1.3 %, *N* = 269). In the same period, seropositivity rate of sarcoptic mange among captured wolves in Sweden was 7.6 % (SE = 2.5 %, *N* = 112). Of the total 21 wolves considered to have sarcoptic mange at necropsy, ten were euthanized due to mange-caused emaciated status (starvation and alopecia), two were illegally shot, two were shot during licence hunt, one was culled to prevent predation on livestock, one was euthanized after a traffic accident and five were found dead. Of the five wolves found dead, four died due to mange-related starvation and one after a traffic accident. In summary, 15 (5.6 %) of the known wolf mortalities reported by the Swedish National Veterinary Institute (SVA) in Sweden between 2003 and 2013 were either euthanized or died because of sarcoptic mange. A systematic serological survey was not carried out on the necropsied wolves, thus it was not possible to include the necropsy data in to the statistical analysis.

The age distribution of the 19 wolves with known age and lesions of mange at necropsy was nine pups (5 to 12 months old; 47.3 %), seven juveniles (1 and 2 years old 36.8 %), and three adults (2 to 6 years old 15.8 %). The majority (9 and 7 respectively) of these wolves came from two different wolf territories. In one territory the adult pair and two 9 months old pups were captured. At capture, the adults were seropositive, while the pups were seronegative. However, the pups were euthanized at the age of 15 and 19 months due to lesions from sarcoptic mange [22]. One of them was seropositive at necropsy, while the serum of the other was not tested.

### Probability of seropositive samples

All 56 pups captured within their parental territories, were seronegative and did not show any lesions of sarcoptic mange. They were therefore excluded from the statistical analysis. Annual mean seroprevalence of the adult population was 19.9 % (SE 6.5 %).

**Fig. 2:**
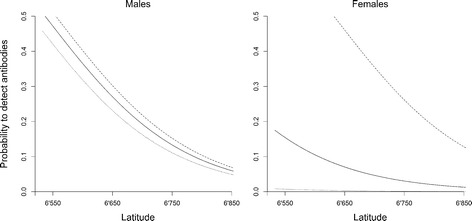
Probability of seropositive serum samples from captured wolves depending on sex, pack size and latitude. Dotted line for pairs, solid line for a pack of six wolves and dashed for a pack with eight wolves. Both figures show the back transformed and averaged predictions of the combined model with e^(Combined/(1+Combined)^

The model combining the population-level predictor latitude and the individual-based interaction of sex and pack size was the most parsimonious (Table 3). According to the relative AICc weight ratio, it had 1.5 higher evidence than the next-best model, which included the wolf territory density. Both models passed model validation with r_s_ > 0.8 (Table 3), but r_s_ is not penalized for additional variables. By adding body condition or removing pack size, the models decreased in evidence but remained within the AICc cut off value (Table 3). Latitude correlated negatively with the likelihood of finding seropositive adult wolves for all combinations of sex and pack size except for females in pairs (Fig. 2a, Table 4). The probability of finding seropositive adult wolves was 6.1 to 8.2 times higher in the southern part (eg UTM 6 600 000, seroprevalence males 36.5 %, females 10.5 %) in packs with 6 wolves (mean size of reproducing packs), as compared to the northern edge of the wolf distribution. The probability of finding seropositive adult males increased linearly with pack size, from 6.6 % seroprevalence in pairs to 38.9 % seroprevalence in packs of eight wolves in the southern part of the study area. For adult females in the same area, the model predicted a seroprevalence of 37.1 % for packs of eight wolves. No adult female from a pair without pups was seropositive, the model predicts a seroprevalence < 1 %.

**Table 3:** Model selection for predictors of seroprevalence of sarcoptic mange in adult wolves captured in Scandinavia

Model	Parameters	K	AIC_c_	ΔAIC_c_	ω_*i*_	r_s_(SE)
Combined	Lat + Sex * Pack Size	5	78.16	0	0.38	0.86(0.02)
+ Density	Lat + Sex * Pack Size + Territory Density	6	79.00	0.84	0.24	0.86(0.03)
+ Body Condition	Lat + Sex * Pack Size + Body Condition	6	79.04	0.88	0.24	0.59(0.03)
– Pack Size	Lat + Sex	3	80.57	2.41	0.12	0.62(0.03)
Submodel intrinsic	Sex * Pack Size	4	83.36	5.20	0.01	-
Submodel density	Lat + Pack Size	3	84.83	6.67	0.01	-
Density	Lat + Long + Pack Size + Territory Density	4	88.47	10.31	0	-
Intrinsic	Sex * Pack Size + Body Condition + Age + Repro	6	90.41	12.25	0	-
Null model	1	1	92.00	13.84	0	-

Top models are validated by k-fold cross validation (r_s_). Lat / Long: Latitude and longitude of the territory centre point, Repro: Reproducing or non-reproducing pair, Pack Size: Number of wolves within the territory, Territory Density: Mean Euclidian distance to the next three territory centre points, Body Condition: Individual residual distance to the linear regression line of log body weight and log body length. Presented are the two main models (Intrinsic, Density), the top models for each variable group, the model combining the top submodels, the combined model with variables ranking within the cut off in the variable group model selection and the null model

**Table 4:** Top model estimates explaining seroprevalence of sarcoptic mange in adult wolves captured in Scandinavia

Factors	Estimate	SE	Lower CI	Upper CI
Intercept	−57.16	26.3	5.56	108.76
Lat	- 0.88	0.39	- 1.64	- 0.11
Sex _*female*_	- 6.12	3.67	- 13.31	1.07
Pack Size	0.05	0.15	- 0.24	0.34
Sex _*female*_ * Pack Size	0.76	0.46	- 0.15	1.66
Territory Density	0.02	0.02	- 0.01	0.05
Body Condition	−4.09	3.53	- 11.01	2.82

Estimates are averaged among the four top models. Lat: Latitude of the territory centre point, Pack Size: Number of wolves within the territory, Territory Density: Mean Euclidian distance to the next three territory centre points, Body Condition: Individual residual distance to the linear regression line of log body weight and log body length

**Fig. 3:**
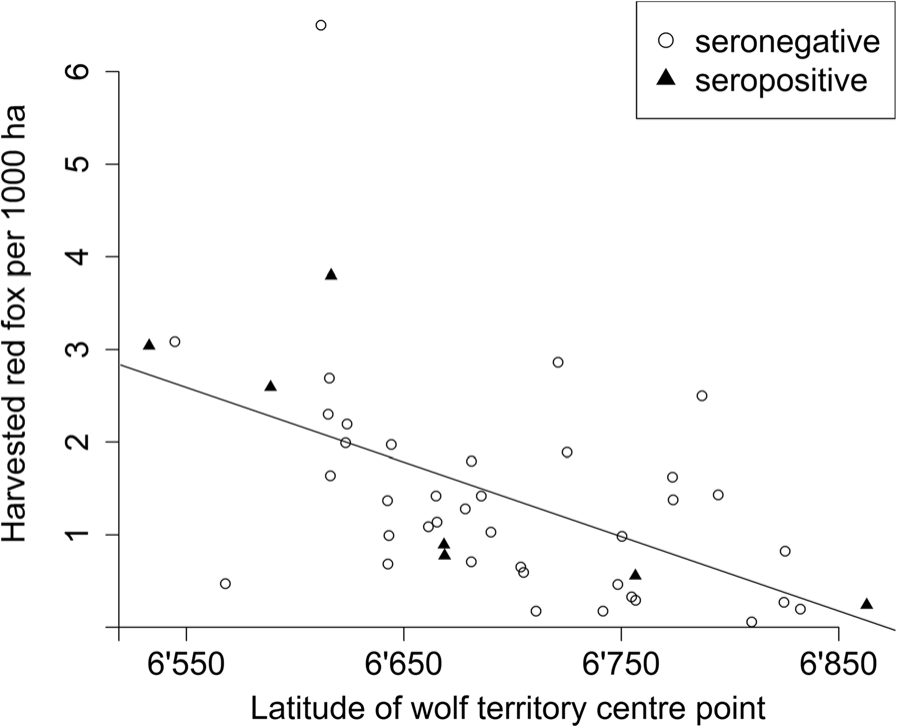
Harvested red fox per 1000 ha for each sampled Swedish wolf territory along the latitudinal gradient (*N* = 49). Circles indicate territories with only seronegative samples, and triangles indicate territories with at least one seropositive sample in the respective years

Red fox harvest data was available for 49 Swedish territory centre points throughout the study period. There was no difference in red fox densities between territories with seropositive wolves (harvested red fox per 1000 ha ± SE = 1.6 ± 0.5) and territories with only seronegative wolves (harvested red fox per 1000 ha ± SE = 1.4 ± 0.2) (t = −0.31, df = 9.4; *p* = 0.77). However, the fox harvest decreased along the geographical gradient from the southern to the northern most territories (Fig. 3; r^2^ = − 0.59, df = 43, *p* < 0.001).

## Discussion

The higher probability to find Scandinavian wolves seropositive to sarcoptic mange in their southern population range is consistent with a higher red fox density and higher habitat productivity resulting in smaller wolf territory size as described by J Mattisson, et al. [23] in these southern latitudes. The positive association between habitat productivity and frequency of mange on wolves is also reported from Yellowstone National Park [7]. In northern Spain reported seroprevalence (20 %, *N* = 17/88) is two times higher than the seropositivity rate in Scandinavia. This difference may be related to higher wildlife densities and more contact to livestock and domestic dogs in Spain [9, 24]. However, we could not find a direct relationship between red fox density and the occurrence of seropositive individual wolves. Reasons for this could be that red fox bag statistics do not represent the density of infectious red foxes especially after a regional mange outbreak [25], and the occurrence of infected wolves after a mange peak in the red fox might be delayed [9]. Bag statistics in general have been shown to be an appropriate index to estimate population densities [25, 26].

Consistent with previous reports [7, 9], we found wolves that had recovered from sarcoptic mange and also cases of coexistence of seropositive wolves, both with and without clinical symptoms, with seronegative and healthy wolves within the territory. But we also had other cases with high mortality on the pack scale. Co-infection with other diseases in this population such as canine distemper virus (CDV), causing immune depression, could lead to increased mortality [27, 28]. The risk for a host to get infected may relate to the initial mite load. Possibly a threshold needs to be reached to overwhelm the host’s immune system, resulting in a high total mite load and increased probability to spread the disease. In humans, *S. scabiei* transmission usually occurs in close body contact and patients carrying >100 adult female mites are much more likely to spread the disease than patients with lower mite rates [29]. Á Oleaga, et al. [9] reported mite rates of <100 isolated mites on wolves and a negative relation of isolated mites to the area of alopecic skin, suggesting a certain ability to control the mite development. The hypersensitive reaction leading to pruritus and alopecia might be present even if the mite rate is low [6]. In Yellowstone National Park, within-pack transmission occurs. Almberg et al. [30] reported a 61 % increased risk of individual infection with a 10 % increased prevalence within packs and that mortality hazards increased with the proportion of infected pack members or ambient temperatures above average but decreased with increasing prey availability.

The large proportion of young wolves among the infested individuals at necropsy demonstrates their sensitivity once infested with sarcoptic mange. In several North American wolf populations, sarcoptic mange decreased pup survival [8, 31, 32]. In Scandinavia, a large part of the mange-infested subadult wolves at necropsy came from only two territories and among the captured individuals, none of the pups were seropositive. We cannot rule out low detectability of infested pups due to high mortality before sampling during winter. In this case we would expect some seropositive survivors and, a negative relation between seroprevalence in adults and pack size. This is not supported by our findings. A seasonal cycle on wolves with lesions of mange has been observed in the Yellowstone National Park with peaks in November on population scale and in January on pack scale [30]. Our samples are collected between December and March.

During winter, Scandinavian wolf pups may be separated from their parents for a large part of the time even though they do not leave the territory until dispersal and do not generally contribute to the food acquirement of the family group [33]. For these wolf pups, exposure to live or recently-killed mange-infested red fox is likely to be very low. Experimentally measured seroconversion in domestic dogs and red foxes takes up to 1–5 weeks post-infection and 1–3 weeks from onset of clinical signs [3, 34] and persists for 1 to > 4.5 months after successful treatment in domestic dogs [35]. Infected pups, 7 to 10 months old at capture, would likely be detected by the ELISA. We suggest that wolf pups in Scandinavia are less exposed to *S. scabiei* than adults.

In contrast to other studies on sarcoptic mange and wolves [24, 30, 31], we found a lower probability of seroprevalence in adult females of small packs. Sex differences in the humoral response to sarcoptic mange are found in Iberian ibex *Capra pyrenaica hispanica* infested with *S. scabiei* and higher Immunoglobulin G (IgG) levels in females than males [36]. Higher IgG levels in females could lead to a longer duration of measurable antibody response potentially resulting, in contrast to our findings, in a higher detectability. Considering the observed recoveries, if higher antibody titres would lead to a faster clearance of the mite, detectability could be lower. IgG levels are currently not available for this data set and IgE or IgA levels measured on a subset of the wolf samples did not differ between males and females nor between mange seropositive and seronegative sera [21, 37] The observed positive correlation of mange prevalence in adult female wolves with pack size may be related to increased susceptibility to parasites during parturition and lactation [6].

Due to the high sociality and the monogamous mating system, sex differences in behaviour of breeding wolves are expected to be small, except during the early stage of nursing the pups [38]. Wolves display sexual dimorphism as male wolves are 20–30 % heavier than females [16, 39] and have a higher food intake [33, 40]. Exposure might be higher for males if they spend more time on carcasses shared with infested red foxes. The breeding wolf pairs in Scandinavia move together and use their territory equally during most of the year [23, 41]. However, we do not know if individual male and female wolves have different predation patterns on red fox or domestic dogs. Considering the assumption of restricted mite development on wolves and the heterogenic patterns on pack scale, individual variation regarding infection paths and immunological response might play an important role. Our small sample size, not allowing us to control for individual variation, might bias model based estimates on individual factors such as sex. Individuals to be captured where chosen according to the research- or management questions and sampling was opportunistic. However, to our knowledge this is the largest serological survey for sarcoptic mange on free-ranging wolves. This is a retrospective study, clinical evaluation of the captured animals was not standardized, did not classify mange lesions, or reports were incomplete. For seven of the 20 seropositive wolves, lesion consistent with sarcoptic mange (alopecia) was noted at capture. For five seropositive wolves normal fur was noted at capture and for eight seropositive wolves information was missing. Two wolves with seropositive test results had clinical signs (alopecia) at capture. No skin biopsies or skin scrapings have been conducted. We therefore were not able to define a nominal reference gold standard and do not report sensitivity and specificity of the ELISA. We confirmed the diagnosis using two ELISAs with different antigens in parallel and Western Blot in addition. Previous studies reported sensitivity and specificity of 92 and 96 % respectively, on domestic dogs with acute sarcoptic mange and 95 and 98 % in chronic cases applying the crude antigen [42]. For the same ELISA, sensitivity and specificity of 95 and 83 % respectively are reported on wild Norwegian red fox [43]. We assume similar values for the wolves, considering the close phylogenetic relation of the domestic dog and the red fox to the wolf. The ELISA using the MSA-1 along with the crude antigen is today used by SVA to diagnose sarcoptic mange on domestic dogs but not validated yet. We consider the three analyses to complement each other and assume sensitivity and specificity at least as high as in the validation studies.

## Conclusions

Although continuously present, we do not consider sarcoptic mange as a significant factor of the sustainability and recovery of the Scandinavian wolf population. Wolf pups are less exposed to the parasite and mange dynamics in the wolf population are not related to the local density of wolf territories. Heterogenic seroprevalence distribution within the packs and repeated observed recovery suggest an effective host - parasite response and a restricted wolf-to-wolf transmission. This disease cannot be regarded as a potential factor controlling population growth at this stage of population development. However, devastating mortality may occur on individual pack scale as a result of this parasite. The higher probability of sarcoptic mange in the southern part of the wolf distribution is probably related to landscape factors and red fox population dynamics. Sarcoptic mange could potentially become more important along with a southern expansion of the Scandinavian wolf population. Future research should address the individual humoral response of wolves and other canids to *S. scabiei*. In Scandinavia, the scale of research should include the entire carnivore guild for a more complete view on the dynamics and effects of this important disease.

## Methods

### Study area

**Fig. 4:**
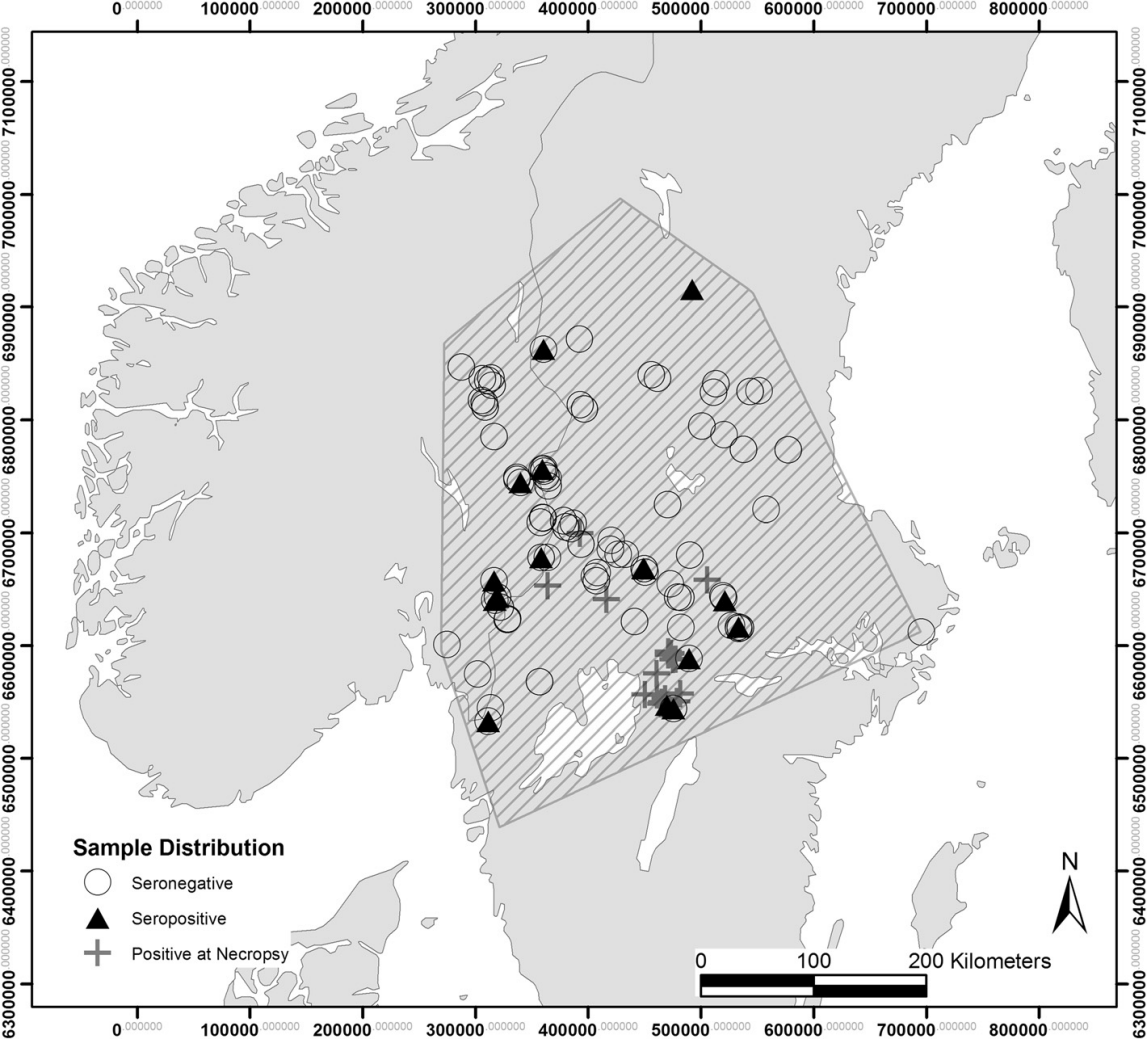
Study area. Distribution of the Scandinavian wolf population (hatched), pack centre points of captured territorial wolves or pups displayed as seropositive (*N* = 20) or negative (*N* = 178) according to the ELISA and the location of collected wolves with lesions of mange at necropsy (*N* = 21). Due to data collection of several animals at the same location, symbols might be overlapping

The Scandinavian wolf population is located in central Sweden and south-eastern Norway (between 59 and 62°N, and 10–15°E) (Fig. 4) [17]. The area is primarily covered by managed forest stands of Norway spruce *Picea abies* and Scots pine *Pinus sylvestris* and wet lands, agricultural areas and settlements covering minor areas, primarily in the southern part of the wolf range [23]. The human population density is less than 1/km^2^ in large areas of the main wolf range [16]. The climate is continental with average temperatures of 15 °C in June and −7 °C in January and the area is snow covered from December to March with an average depth of 30–60 cm [41]. The main prey species are moose *Alces alces* and roe deer *Capreolus capreolus,* the latter mainly in the southern range of the wolf area. Other prey species represent a minor proportion of the diet [33, 44].

### Serum collection and analysis

A total of 198 serum samples from 145 individual free-ranging wolves of 54 different packs in Sweden and Norway were analysed. All wolves were immobilized by darting from a helicopter during winter (December-March) between 1998 and 2013 as part of a long term joint Scandinavian research project or by national management, both following standard capture procedures [45] and in accordance with the NC3Rs ARRIVE guidelines for reporting animal research [46]. Captured animals were tagged with VHF or GPS collars and ear tags or microchips and the identity further confirmed by DNA- and pedigree analysis [15]. To change radio collars or for translocations 38 individuals were recaptured 1–3 times (Table 5). A sampling event per territory was defined as one or more wolves captured per winter within a pack, including recaptures. Sampled animals were sexed (104 males, 94 females), weighed and measured. As a proxy for body condition, we used the residual distance from each individual to the linear regression line of log body weight and log body length (nose to base of the tail) estimated from all individuals in the data set [47]. This residual index was no longer correlated to body length (*r* = −0.01, df = 104, *p* = 0.94). The animals were grouped into pups (<1 year old) and adults. The age was estimated from tooth wear in adults and pups were identified by the growth zone on the radius and ulna [48]. Based on movement data from GPS-collared wolves, pups were assumed to be born in early May [49]. The age of the sampled wolves ranged from 7 to 10 months for pups and over 10 years for adults. Age was adjusted if pedigree analysis could prove the year of birth. Clinical evaluation of the captured wolves was not standardized but anomalies, such as previous injuries, broken teeth or mange lesion, are mentioned on the capture form. Blood was collected from the cephalic vein using 8 ml sterile, evacuated serum-separator tubes with gel and clot activator (Venosafe™, Terumo Europe N.V, Leuven, Belgium). Serum was separated by centrifugation at 1500 rpm for 15 min the same day and kept frozen until shipment to the laboratory (Department of Virology, Immunobiology and Parasitology, SVA Uppsala, Sweden).

**Table 5:** Age and recaptures among the captured individuals

		Recaptures			
	Captured individuals	1	2	3	Total samples
Pups	56				56
Pups, recaptured as adults		13	5	1	19
Adults	89	25	6	3	123
Total	145	38	11	4	198

Pups are < 1 year old, adults > 1 year old

Serum samples were analysed by running two parallel ELISAs using a crude (*S. scabiei* var. *vulpes* extract) antigen according to Bornstein et al. 1993 [34], modified by a change of the substrate from 5-amino-2-hydroxybenzoic to 3-3-5 tetramethylebenzidin (TBM) and a recombinant major *S. scabiei* var. *vulpes* antigen (MSA-1) respectively [50]. In each series, samples were tested in duplicates and positive and negative control samples from dogs were included. Optical density (OD) was measured at 450 nm with a multiscan EX (Thermo Labsystems, Vantaa, Finland). In order to get an OD value relative to the positive control, mean OD values for each duplicate were calculated and the mean OD of a blank (PBS-T instead of a sample) subtracted. For valid results, positive control OD values had to be between 0.8 and 1.6 for the crude antigen and between 0.7 and 2.0 for the MSA-1 antigen. OD values of the samples were divided by the OD value of the positive control. Positive results should reach > 0.3 for the crude antigen and > 0.5 for the MSA-1, relative to the positive control. Doubtful results were defined as 0.2–0.299 by the crude antigen and 0.35–0.499 by the MSA-1 antigen, OD values below 0.2 and 0.35 respectively were considered to be negative.

Western Blot as previously described [3], was used to confirm all seropositive samples, all doubtful samples and all samples with different results between the two ELISAs. In brief, the sarcoptes proteins in the crude antigen extract were separated by their atomic weight (kDa) on a nitrocellulose membrane. The samples were exposed to the proteins. When positive, the antibodies in the samples bound with the protein at 164 kDa or 147 kDa and 105 kDa. Samples with doubtful or positive ELISA values but confirmed positive by Western Blot were considered as seropositive.

The proportion of seropositive samples are presented with confidence intervals estimated by the Wilson score method [51].

### Necropsy reports

After 2002, all dead wolves found in Sweden have been delivered for standard necropsy at SVA. Serology as described above (*N* = 15), microscopy (*N* = 4) or both (*N* = 2) were part of the standard necropsy if clinical signs of sarcoptic mange were detected. We compared observed seropositivity from captured Swedish wolves from 2003 to 2013 with the necropsy data.

### Population data

The annual monitoring of the Scandinavian wolf population for the entire study area was based on snow tracking, DNA-analyses of scats and radio/GPS collar data [52]. This monitoring provided, for each winter, the total number and geographical distribution of established pairs and packs and estimates of pack- and population size. We expressed wolf density at two spatial scales. Within wolf territories, pack size represented the number of wolves per territory and ranged from one to ten wolves. The samples were classified as from single wolves, from pairs (two scent marking animals of opposite sex) or packs (one or two scent marking animals and pups or yearlings). Wolves older than two years within the parental territory and both parents present have until the end of the study period not been detected by the monitoring in Scandinavia [17]. At the landscape level, we used distance to neighbouring packs as a proxy for territory density. We estimated the territory densitiy for each wolf territory and for each winter as the average of the Euclidean distances between its centre point and the centre points of the three nearest neighbouring wolf territories using the spatstat package [53] in R 3.0.3 [54]. We estimated the centre point coordinates by averaging VHF or GPS collar locations of territorial adult wolves, and DNA collection sites for wolf territories without radio-collared individuals.

We used latitude as a habitat productivity proxy and checked for longitude effects. As a proxy of red fox density for each wolf territory, we used the yearly number of red fox harvested per 10 km^2^. These bag statistics were provided by the Swedish Association for Hunting and Wildlife Management on the hunting management unit level (mainly corresponding to a municipality) in Sweden. Mange lesions or other qualitative measures of the shot red fox were not reported. Using a two sample *t*-test, we tested if occurrence of seropositive wolves was related to high red fox densities. We used the red fox harvest record from those hunting management units that contained the centre points of the wolf territories. Accordingly, we tested if the latitude of the centre points was related to the red fox harvest record in the respective hunting management units.

### Modelling seroprevalence

According to our hypothesis we formulated two full models *à priori*: 1) The individual-based model correlated the probability to find a seropositive serum sample to individual-based intrinsic factors; reproductive state, age, body condition, and the interaction of sex and pack size, assuming more physical contact of reproducing females with their pups, as well as a potential negative effect on pup survival with seropositive females. 2) The population-based model correlated the probability to find a positive serum sample to territory density, and to the projected, metric latitude and longitude of the wolf territory. Territory size of Scandinavian wolves increases with latitude, independently of available moose biomass but related to landscape structure and climate [23]. We did not include red fox density in this model because of missing values for the Norwegian territories. The response variable in both models was a binary term with 1 for seropositive and 0 for seronegative wolves, and models were statistically estimated using logistic regression. Due to a low number of events (seropositive) the risk of over-fitted models increases rapidly with every included variable [55]. We formulated sub models with fewer variables and included them in the selection. Within both variable groups sub-models performed better than the full models (Table 3).

All analyses were done using statistical extensions available in R 3.0.3 [54]. Despite the inclusion recaptured individuals we did not include nested random factors to the models. This decision was based on the lack of the among-individual variance to the binary response variable resulting in a negligible effect when included as a random factor [56]. A large proportion of the individuals (107 of 145, Table 5) were sampled only once. In addition, the time interval between capture events was long enough for individuals to change from seropositive and seronegative and vice versa [34, 36]. The variance inflation factor of any continuous variable was <1 indicating no multicollinearity [57] and models did not show outliers (Cook’s distance) [58].

We selected by parsimony for each full model the best sub-model (lowest AICc using the R-package AICcmodvag, no cut off). Then we used a cut-off point of delta AICc = 4 to find the best combination of predictors from both sub-models. Further, we used model-averaging to present the effect sizes of the predictors of the models within the cut-off [59]. Observations with lacking data were omitted before model selection (*N* = 36). Robustness of the selected models was verified by k-fold cross validation. The models were run 100 times on a training set including randomly selected 90 % of the data. Using the estimates of the training set, probabilities of being seropositive were predicted for the remaining 10 %. The predicted values were sorted and grouped into 10 equal-sized, ranked bins. For each bin, the frequency of seropositive individuals was correlated with the rank of the bin using a Spearman rank correlation (r_s_). This process was repeated 10 times and the mean values of r_s_ are presented.

For the modelling process we omitted 36 out of the 198 samples either because data on body condition were missing (*N* = 21), the wolves were dispersing (*N* = 4) or were immigrants from the Finnish-Russian population resident but captured for translocation (*N* = 2). Four samples from one individual were excluded due to unclear results from both ELISA and Western Blot. On average, 9.3 % (SE 1.4 %) of the estimated mean Scandinavian wolf population was sampled each winter (Fig. 1a).
